# miR‐1249‐3p accelerates the malignancy phenotype of hepatocellular carcinoma by directly targeting HNRNPK

**DOI:** 10.1002/mgg3.867

**Published:** 2019-08-20

**Authors:** Hongchun Shu, Jia Hu, Huiqiu Deng

**Affiliations:** ^1^ Department of Gastroenterology, Jiangxi Institute of Gastroenterology & Hepatology The First Affiliated Hospital of Nanchang University Nanchang P. R. China; ^2^ Gastroenterology Department ShangRao People’s Hospital Shangrao P. R. China

**Keywords:** cell events, hepatocellular carcinoma, *HNRNPK*, *miR‐1249‐3p*

## Abstract

**Background:**

microRNAs (miRNAs) have been implicated to play crucial roles in carcinogenesis. *miR‐1249‐3p* was reported to be abnormally expressed in multiple human cancers. However, its biological role and the associated underlying mechanisms in hepatocellular carcinoma (HCC) remain largely unknown.

**Methods:**

*miR‐1249‐3p* expression level in HCC cell lines and normal cell line was measured by quantitative real‐time PCR. Role of *miR‐1249‐3p* on HCC cell proliferation, colony formation, and invasion was examined by cell counting kit‐8 assay, colony formation assay, and transwell invasion assay, respectively. Luciferase activity reporter assay and western blot were performed to validate whether heterogeneous nuclear ribonucleoprotein K (*HNRNPK*) was a direct target of *miR‐1249‐3p*. Effect of *miR‐1249‐3p* on overall survival of HCC patients was analyzed at KM Plotter website.

**Results:**

We found *miR‐1249‐3p* expression level was increased, while HNRNPK expression level was decreased in HCC cell lines compared with normal cell line. Knockdown *miR‐1249‐3p* expression inhibits HCC cell proliferation, colony formation, and cell invasion through regulating *HNRNPK* in vitro. We also showed high *miR‐1249‐3p* expression was a predictor for poor overall survival of HCC patients.

**Conclusions:**

These findings about *miR‐1249‐3p*/*HNRNPK* pair provide a novel therapeutic method for HCC patients.

## 1 INTRODUCTION

Hepatocellular carcinoma (HCC) is a major health threat worldwide (Bray et al., 2018). Most of HCC cases were found in advance stages as it is difficult for early diagnosis (Ghouri, Mian, & Rowe, 2017). Emerging evidence has indicated that HCC tumorigenesis is associated with dysexpression of multiple key genes and signaling pathways (Yin et al., 2016; Zhou, Du, Kong, Zhang, & Chen, 2018). Thus, it is imperative to investigate molecular mechanisms behind HCC tumorigenesis to explore novel biomarkers for cancer diagnosis or treatment.

microRNAs (miRNAs) are reported to regulate majority of human genes, and hence to play a crucial role in cancer initiation and progression, including HCC (Krol, Loedige, & Filipowicz, 2010). For example, Babu et al. found *miR‐148a* expression level was decreased in HCC tissues compared with normal tissues (Babu & Muckenthaler, 2019). Overexpression of *miR‐148a* using *miR‐148a* mimic significantly decreased HCC cell proliferation through targeting transferrin receptor 1 (616740) (Babu & Muckenthaler, 2019). Wu et al. revealed that *miR‐29c‐3p* expression was downregulated in HCC tissues and cell lines in comparison with normal tissues and cell lines (Wu et al., 2019). Force *miR‐29c‐3p* expression inhibited HCC progression in vitro and in vivo through regulating large tumor suppressor gene 1 (603473) (Wu et al., 2019). Chen et al. identified *miR‐195* was downregulated in HCC and correlated with lymph node metastasis and TNM stage (Chen & Wang, 2019). Furthermore, they should overexpression of *miR‐195* could promote HCC cell malignancy behaviors, while the knockdown of *miR‐195* will cause the opposite effects.

*miR‐1249* was previously reported to be abnormally expressed in several human cancers (Chen et al., 2019; Fang, Li, Xu, Hui, & Li, 2018; Ye et al., 2017). For instance, *miR‐1249* was found upregulated expression in glioma tissues and cell lines, and overexpression of *miR‐1249* enhanced glioma progression in vitro and in vivo through targeting adenomatous polyposis coli 2 (Fang et al., 2018). In colorectal cancer, *miR‐1249* was reported as a transcriptional target of P53 (191170) to suppress tumor growth, metastasis, and angiogenesis (Chen et al., 2019). Importantly, *miR‐1249* expression was found could activated by Hedegehog signaling pathway to stimulate HCC progression (Ye et al., 2017). Nevertheless, the biological role and molecular mechanisms of *miR‐1249‐3p* in HCC remain to be elucidated.

In current study, we measured *miR‐1249‐3p* expression level in HCC cell lines and normal cell line. HCC cell proliferation, colony formation, and invasion after synthetic miRNAs transfection were examined by cell counting kit‐8 assay, colony formation assay, and transwell invasion assay, respectively. Luciferase activity reporter assay and western blot assay were conducted to validate heterogeneous nuclear ribonucleoprotein K (600712, *HNRNPK*) as a direct target of *miR‐1249‐3p*. Effect of *miR‐1249‐3p* expression on overall survival of HCC patients was analyzed using bioinformatic tool.

## 2 MATERIALS AND METHODS

### 2.1 Cell line and cell culture

HCC cell lines (Huh7 and Hep3B) and normal liver cell line L02 obtained from Cell Bank of Chinese Academy of Sciences (Shanghai, China) were incubated at Dulbecco's modified Eagle's medium (DMEM; Thermo Fisher Scientific, Inc.) supplemented with 10% fetal bovine serum (FBS, Thermo Fisher Scientific, Inc.) and 1% penicillin/streptomycin (Thermo Fisher Scientific, Inc.) at a 37°C humidified incubator containing 5% CO_2_.

### 2.2 Cell transfection

miRNA inhibitor (miR‐inhibitor) and the corresponding negative control (miR‐NC) were purchased from GenePharm. Small interfering RNA targeting *HNRNPK* (siR‐*HNRNPK*) and the negative control (siR‐NC) were also obtained from GenePharm. Cell transfection was conducted using Lipofectamine 2000 (Thermo Fisher Scientific, Inc.) according to the manufacturer's instructions.

### 2.3 RNA extraction and quantitative real‐time PCR (qRT‐PCR)

Total RNA from cultured cells were extracted using Trizol reagent (Thermo Fisher Scientific, Inc.) and quantified using Nanodrop‐2000 (Thermo Fisher Scientific, Inc.). Complementary DNA (cDNA) was synthesized from RNA using PrimeScript RT Reagent (Takara). qRT‐PCR was performed at ABI 7500 system (Applied Biosystems) using SYBR Premix Ex Taq II (Takara). Primers used were as follows: *miR‐1249‐3p* forward, 5′‐ACACTCCAGCTGGGACTTCTTCCCCCCCTT‐3′ and reverse, 5′‐CTCAACTGGTGTCGTGGAGTCGGCAATTCAGTTGAGTGCGGGAA‐3′; *U6 snRNA* forward, 5′‐CTCGCTTCGGCAGCACA‐3′, and reverse, 5′‐AACGCTTCACGAATTTGCGT‐3′. The procedures were as follows: 1 cycle of 95°C for 10 min, followed by 40 cycles of 95°C for 15 s and 60°C for 1 min.

### 2.4 Protein isolation and western blot

Total protein from cultured cells was isolated using RIPA lysis buffer (Beyotime) and quantified with BCA protein kit (Beyotime). Equal amount of protein sample was isolated at 10% SDS‐PAGE and then transferred to PVDF membrane. Membranes were blocked with fat‐free milk, and then incubated with primary antibodies: rabbit anti‐HNRNPK: ab52600, rabbit anti‐N‐cadherin (608541): ab76011, rabbit anti‐Vimentin (193060): ab92547, rabbit anti‐E‐cadherin (192090): ab40772, rabbit anti‐GAPDH (138400): ab181602 (all from Abcam). After washed with TBST, membranes were incubated with horseradish peroxidase‐conjugated goat anti‐rabbit secondary antibody (ab6721, Abcam). Band signals were developed using BeyoECL kit (Beyotime) and analyzed with Image J 1.42 (NIH).

### 2.5 Cell counting kit‐8 (CCK‐8) assay

Cells were seeded into 96‐well plate with the density of 5 × 10^4^ cells/well. 0, 24, 48, and 72 hr after seeding, CCK‐8 reagent (Beyotime) was added to each well and further incubated for 4 hr. Optical density at 450 nm was recorded to assess cell proliferation.

### 2.6 Colony formation assay

Cells were plated in six‐well plates at the density of 500 cells/well and incubated for 2 weeks. Colonies formed were fixed with methanol and stained with crystal violet. Colonies number was counted from at least five independent fields under microscope.

### 2.7 Transwell invasion assay

Cell invasion ability was assessed using Transwell invasion assay. 3 × 10^5^ cells in DMEM were plated to Matrigel (BD Biosciences) precoated upper chamber, and DMEM containing FBS was added to lower chamber. Twenty‐four hours after incubation, noninvaded cells removed by cotton swab, while the invasive cells were fixed with methanol and stained with crystal violet. Invasion cell numbers were counted under microscope.

### 2.8 Luciferase activity reporter assay

TargetScan v_7.2 (http://www.targetscan.org/vert_72/) was employed to predict the potential target of *miR‐1249‐3p* and we found *HNRNPK* was a possible target. The wild‐type (wt) 3′‐UTR (NM_001318186.1) was amplified from genomic DNA obtained from HCC cells and inserted into pMIR‐REPORT (Promega) to obtain HNRNPK‐wt. Site‐direct mutagenesis kit (Takara) was used to generate mutant *HNRNPK* luciferase vector version (HNRNPK‐mt). Cells were cotransfected with HNRNPK‐wt or HNRNPK‐mt and miR‐inhibitor or miR‐NC using Lipofectamine 2000. Forty‐eight hours after cotransfection, relative luciferase activity was measured using Dual‐luciferase activity reporter assay system (Promega).

### 2.9 Kaplan–Meier curve analysis

Kaplan–Meier plotter (www.kmplot.com, Nagy, Lánczky, Menyhárt, & Győrffy, 2018) was employed to analyze *miR‐1249‐3p* expression on overall survival of HCC patients. Cut‐off value was auto selected in the algorithm. Log‐rank test was used to analyze difference in groups.

### 2.10 Statistical analysis

Data were analyzed at SPSS 19.0 (SPSS Inc.) and then presented as mean ± *SD*. Statistical analysis was analyzed using two‐tailed Student's *t* test or one‐way ANOVA and Tukey’s post hoc test. Differences were considered as statistically significant when *p* < .05.

## 3 RESULTS

### 3.1 Upregulation of *miR‐1249‐3p* in HCC

**Figure 1:**
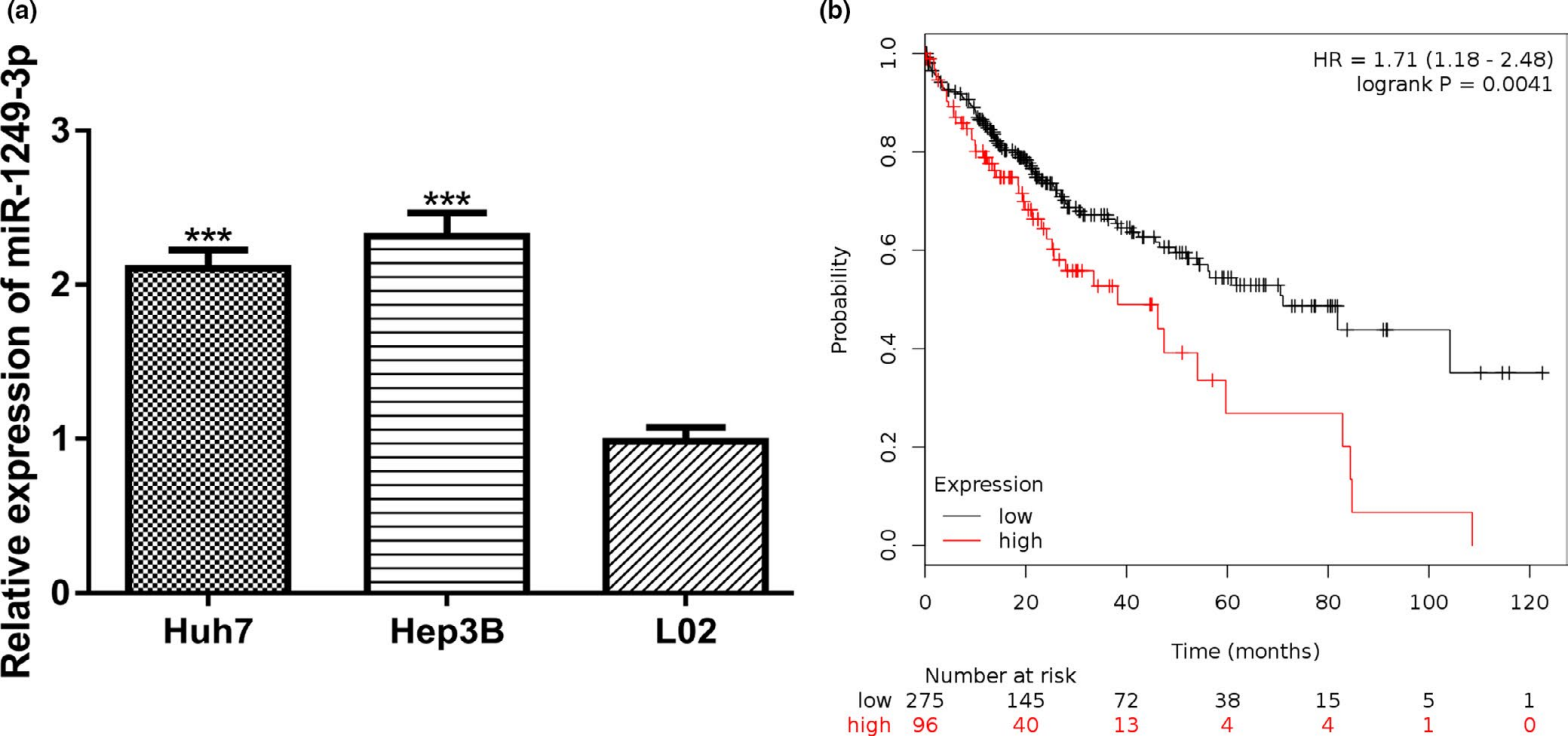
Upregulation of miR‐1249‐3p in HCC. (a) qRT‐PCR to measure miR‐1249‐3p expression in HCC cell lines (Huh7 and Hep3B) and normal liver cell line L02. (b) High miR‐1249‐3p was a predictor for poor overall survival of HCC patients as analyzed at Kaplan–Meier plotter website. HCC, hepatocellular carcinoma; miR‐1249‐3p, microRNA‐1249‐3p; qRT‐PCR, quantitative real‐time PCR; ***p < .001

qRT‐PCR revealed mature *miR‐1249‐3*p expression level was significantly increased in HCC cell lines (Huh7 and Hep3B) compared with normal liver cell line L02 (Figure 1a). Moreover, we found high *miR‐1249‐3p* expression was a predictor for poor overall survival of HCC patients (Figure 1b).

### 3.2 ***miR‐1249‐3p* downregulates *HNRNPK* expression by 3**′**‐UTR binding**

**Figure 2:**
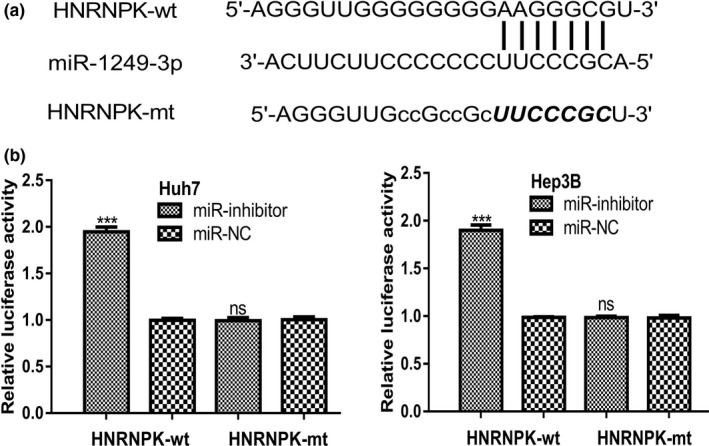
HNRNPK was a target of miR‐1249‐3p. (a) Binding site between miR‐1249‐3p and 3′‐UTR of HNRNPK. (b) Luciferase activity was increased by miR‐inhibitor in cells transfected with HNRNPK‐wt. HCC, hepatocellular carcinoma; HNRNPK, heterogeneous nuclear ribonucleoprotein K; miR‐1249‐3p, microRNA‐1249‐3p; miR‐inhibitor, miR‐1249‐3p inhibitor; miR‐NC, negative control miRNA; mt, mutant; UTR, untranslated region; wt, wild‐type; ***p < .001

TargetScan predicted *HNRNPK* was a possible target of *miR‐1249‐3p* (Figure 2a). To validate whether *miR‐1249‐3p* could bind with the 3′‐UTR of *HNRNPK*, luciferase activity reporter assay was conducted. We found miR‐inhibitor introduction increased luciferase activity of cells transfected with HNRNPK‐wt but not HNRNPK‐mt (Figure 2b).

### 3.3 Inhibition of *miR‐1249‐3p* decreased HCC cell proliferation, colony formation, and cell invasion

**Figure 3:**
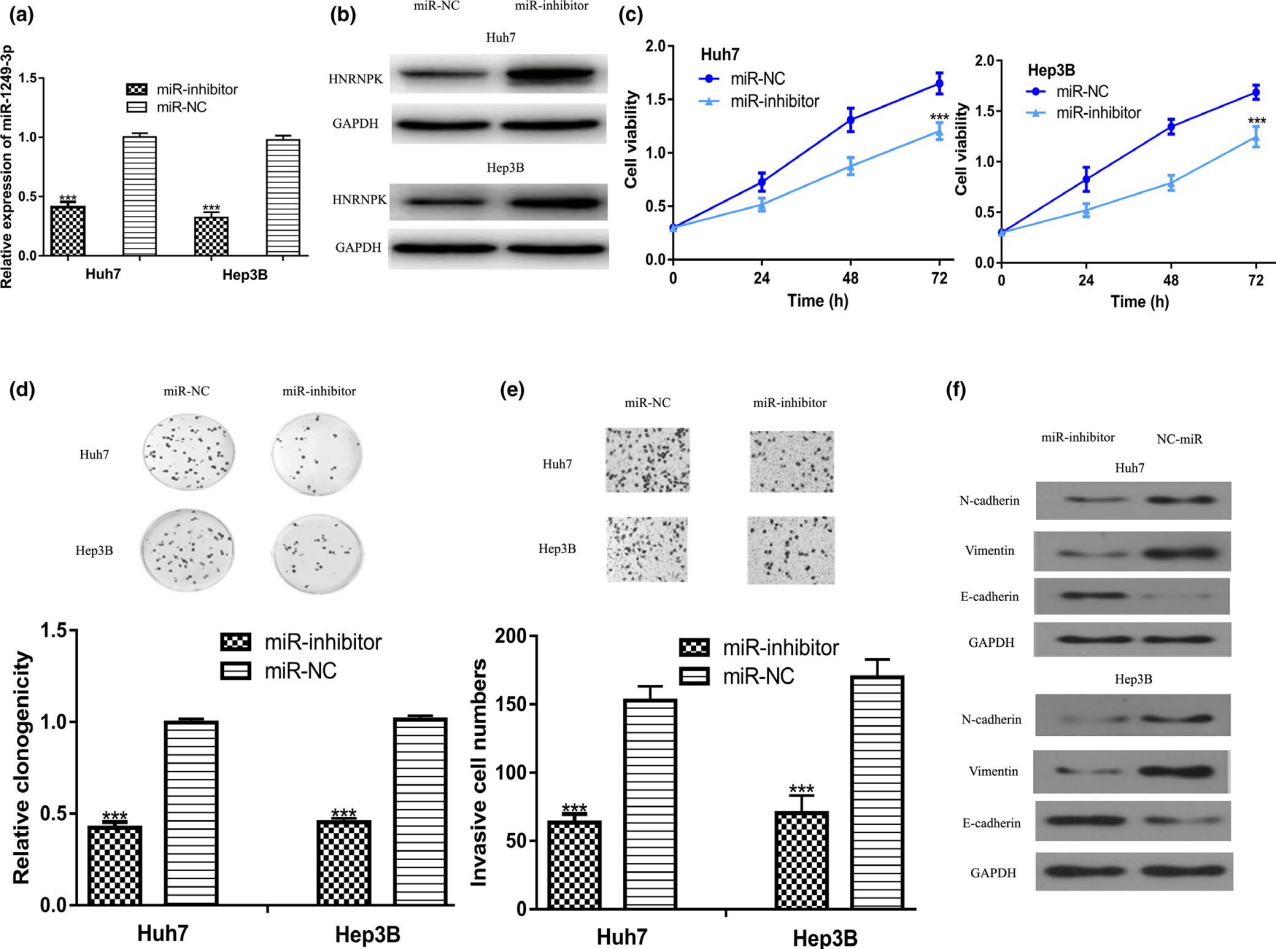
Knockdown of miR‐1249‐3p inhibits HCC cell growth, invasion, and EMT. (a) miR‐1249‐3p expression, (b) HNRNPK expression, (c) Cell proliferation, (d) Colony formation, (e) Cell invasion, and (f) N‐cadherin, Vimentin, and E‐cadherin expression in HCC cells transfected with miR‐inhibitor or miR‐NC. EMT, epithelial‐mesenchymal transition; HCC, hepatocellular carcinoma; miR‐1249‐3p, microRNA‐1249‐3p; miR‐inhibitor, miR‐1249‐3p inhibitor; miR‐NC, negative control miRNA; ***p < .001

To investigate the effect of *miR‐1249‐3p* on HCC, loss‐of‐function studies using synthetic miRNAs were conducted. We found miR‐inhibitor transfection significantly decreased *miR‐1249‐3p* expression level compared with miR‐NC (Figure 3a). Western blot showed miR‐inhibitor transfection increased HNRNPK expression in HCC cell lines (Figure 3b). CCK‐8 assay and colony formation assay revealed that downregulation of *miR‐1249‐3p* decreased the growth of Huh7 and Hep3B cell lines (Figure 3c,d). Furthermore, transwell invasion assay indicated the invasion ability of Huh7 and Hep3B cell lines transfected with miR‐inhibitor was inhibited compared with those transfected with miR‐NC (Figure 3e). Decreased expression of N‐cadherin, Vimentin and increased E‐cadherin expression levels after *miR‐1249‐3p* downregulation were found (Figure 3f). Taken together, these results indicated that overexpression of *miR‐1249‐3p* inhibited the progression of HCC.

### 3.4 *miR‐1249‐3p* regulates HCC cell behaviors via targeting *HNRNPK*

**Figure 4:**
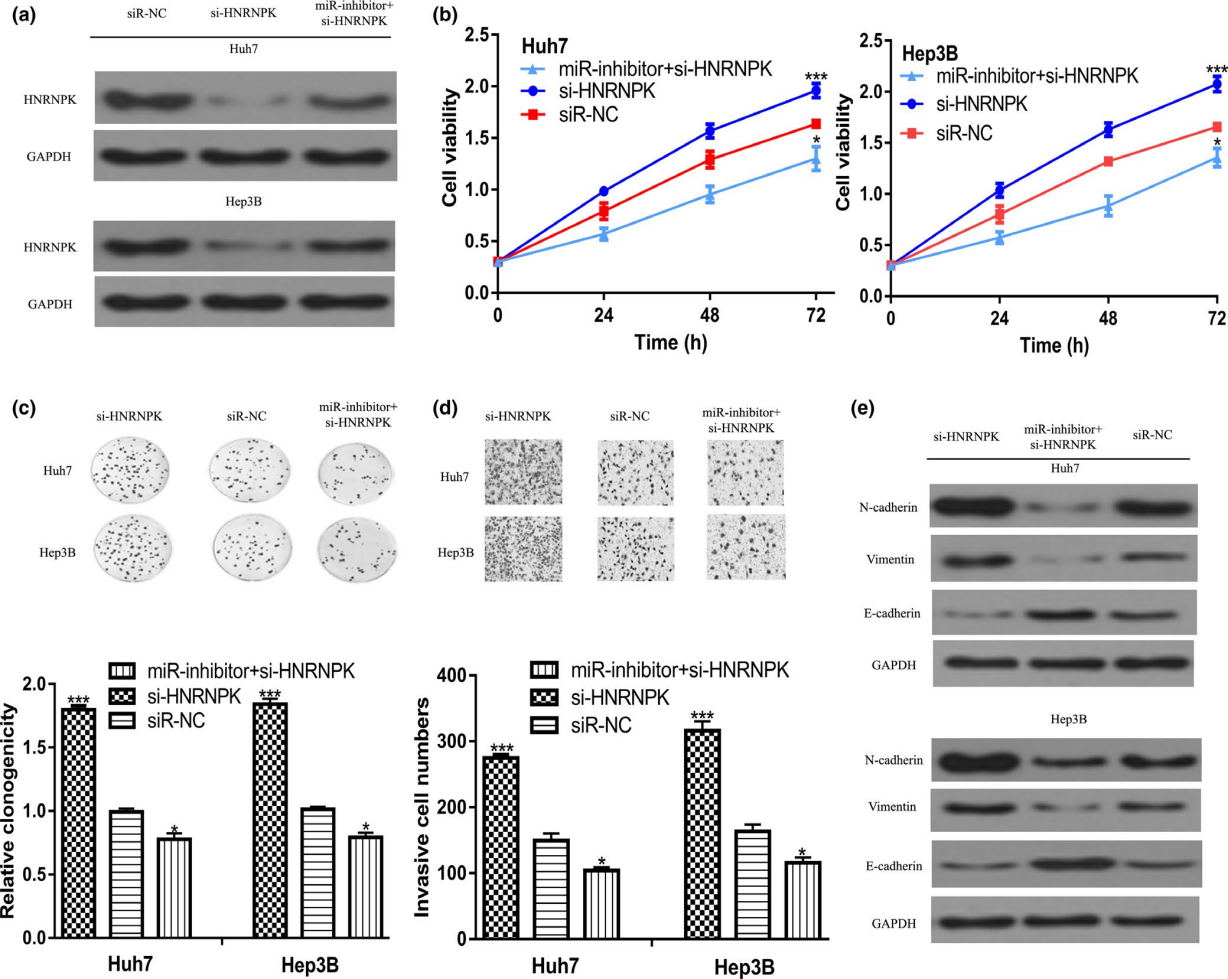
miR‐1249‐3p regulates HCC cell events through targeting HNRNPK. (a) HNRNPK expression, (b) Cell proliferation, (c) Colony formation, (d) Cell invasion, and (e) N‐cadherin, Vimentin, and E‐cadherin expression in HCC cells transfected with si‐HNRNPK, siR‐NC, or si‐HNRNPK and miR‐inhibitor. HCC, hepatocellular carcinoma; HNRNPK, heterogeneous nuclear ribonucleoprotein K; miR‐1249‐3p, microRNA‐1249‐3p; miR‐inhibitor, miR‐1249‐3p inhibitor; si‐HNRNPK, small interfering RNA targeting HNRNPK; siR‐NC, negative control siRNA; ***p < .05, ***p < .001

We then investigate whether HNRNPK was a functional target of *miR‐1249‐3p* by rescue experiments. Introduction of si‐HNRNPK significantly decreased HNRNPK expression level in HCC cells (Figure 4a). CCK‐8 assay and colony formation assay indicated cell growth was increased by si‐HNRNPK (Figure 4b,c). Meanwhile, we showed cell invasion ability was also increased by si‐HNRNPK (Figure 4d). Importantly, we found N‐cadherin and Vimentin expression was increased, while E‐cadherin expression was decreased by si‐HNRNPK (Figure 4e). Moreover, the effect of miR‐inhibitor on cell growth and invasion ability could partially reversed by si‐HNRNPK (Figure 4b–e).

## 4 DISCUSSION

In previous study, *miR‐1249* expression was reported could be activated by Hedegehog signaling pathway in HCC (Ye et al., 2017). However, the downstream targets of *miR‐1249* are largely unknown. In this study, we showed *miR‐1249‐3p* expression was significantly increased in HCC cell lines compared with normal cell line L02. Moreover, we showed high *miR‐1249‐3p* expression was a predictor for poor overall survival of HCC patients, indicating *miR‐1249‐3p* may function as an oncogene in HCC. Loss‐of‐function experiments showed knockdown of *miR‐1249‐3p* inhibited HCC cell proliferation, colony formation, and invasion in vitro. Importantly, we showed several key players in epithelial‐mesenchymal transition (EMT) process could be regulated by *miR‐1249‐3p*. EMT is a process to transfer epithelial cell into mesenchymal phenotype, an indicator for malignancy behaviors of human cell (Tania, Khan, & Fu, 2014).

As small regulator molecules, miRNA regulates target gene expression at posttranscriptional level (Iqbal, Arora, Prakasam, Calin, & Syed, 2018). It has been widely recognized that one gene can be regulated by multiple miRNAs and one miRNA could regulate various genes in a cell context dependent manner (Iqbal et al., 2018). Previous studies identified several targets of *miR‐1249* in human cancers (Chen et al., 2019; Fang et al., 2018). Here, we found *HNRNPK* was a potential target of *miR‐1249‐3p*. HNRNPK is a crucial RNA and DNA binding protein and reported function as a crucial regulator for the progression of human cancers (Barboro, Ferrari, & Balbi, 2014). HNRNPK is involved in multiple biological processes including chromosome remodeling, DNA transcription, RNA processing, and RNA translation (Bomsztyk, Denisenko, & Ostrowski, 2004; Mikula et al., 2006). HNRNPK overexpression can retard gastric cancer cell proliferation and colony formation in vitro and in vivo through regulating p53/p21/CCND1 axis, indicating a tumor suppressive role of HNRNPK (Huang et al., 2017). On the contrary, HNRNPK overexpression was found could increase pancreatic cancer cell proliferation, migration, and invasive (He et al., 2017). Here, we validated *HNRNPK* was a direct target of *miR‐1249‐3p* using luciferase activity reporter assay and western blot assay. Moreover, rescue experiments demonstrated that *HNRNPK* was a functional target of *miR‐1249‐3p*.

In summary, *miR‐1249‐3p/HNRNPK* axis could affect EMT, proliferation, colony formation and invasion of HCC cells. We therefore hypothesized that *miR‐1249‐3p* inhibitor might be a therapeutic agent for HCC but further in‐depth investigations are required.

## CONFLICT OF INTEREST

The authors declare that they have no conflict of interest.
